# Effect of somatometric parameters on the prevalence and severity of varicocele: a systematic review and meta-analysis

**DOI:** 10.1186/s12958-021-00695-3

**Published:** 2021-01-20

**Authors:** Runqing Li, Junjie Liu, Yushan Li, Quanxian Wang

**Affiliations:** 1 The Neonatal Screening Center in Henan Province, The Third Affiliated Hospital of Zhengzhou University, Zhengzhou, 450052 China; 2 Henan Human Sperm Bank, The Third Affiliated Hospital of Zhengzhou University, No.7 Front Kangfu Street, Er’qi District, Zhengzhou, 450052 People’s Republic of China

**Keywords:** Meta-analysis, Varicocele, Age, Height, Weight, Body mass index

## Abstract

**Background:**

Published studies have shown contradictory results regarding the relationship between somatometric parameters and varicoceles. We performed a systematic review and meta-analysis to investigate the possible effects of age, height, weight, and body mass index (BMI) on the presence and severity of varicoceles.

**Methods:**

Databases including EMBASE, MEDLINE, PubMed, Cochrane Library, China National Knowledge Infrastructure (CNKI), Web of Science, and Google Scholar were systematically searched to identify relevant articles published up to March 2020. Two researchers independently identified eligible articles and extracted data. Cochran’s Q statistic and I^2^ statistics were used to assess heterogeneity. Meta-analysis was performed using StataSE 12.0 software (StataCorp LP, USA). Random-effects models were used to obtain the weighted mean differences (WMDs) and 95% confidence intervals (CIs). Publication bias was assessed using Begg’s funnel plot and Egger’s regression test.

**Results:**

The search strategy produced 272 articles, of which 18 articles were eligible according to the inclusion/exclusion criteria. A total of 56,325 patients with varicocele and 1,334,694 patients without varicocele were included in the meta-analysis to evaluate the effect of somatometric parameters on the presence and severity of varicocele. The overall results demonstrated that the presence of varicoceles was significantly associated with height (WMD = 1.41, 95% CI = 1.07 to 1.74, *P* < 0.001) and inversely correlated with BMI (WMD = − 1.35, 95% CI = -1.67 to − 1.03, *P* < 0.001) but not with age (WMD = -0.93, 95% CI = -2.19 to 0.33, *P* = 0.149) or weight (WMD = 0.24, 95% CI = -2.24 to 2.72, *P* = 0.850). The severity of varicocele was inversely correlated with increased BMI but not with age.

**Conclusion:**

The presence of varicoceles was significantly associated with height and inversely correlated with BMI.

## Strengths and limitations of this study

- This meta-analysis included 56,325 patients with varicocele and 1,334,694 patients without varicocele
- The sample size was large enough to draw a reliable conclusion.
- Stratified analyses by study design and ethnicity were also performed in this meta-analysis
- We also investigated the effect of age and BMI on the severity of varicocele.

## Introduction

Varicocele, an abnormal dilation of the pampiniform venous plexus in the scrotum, is the most common surgically correctable cause of male infertility [1–4]. The prevalence of varicocele is 15–20% in the general population, 21–41% in men with primary infertility, and 75–81% in men with secondary infertility [1, 3, 5–8]. The exact mechanism of varicocele development has not been fully clarified. According to existing theories, varicocele is considered to be related to various factors resulting in abnormal dilation of the pampiniform venous plexus and venous drainage [7].

Previous studies have investigated the association between somatometric parameters and the prevalence and severity of varicocele. However, there are contradictory data regarding the relationship between somatometric parameters and the prevalence and severity of varicocele. Some studies have suggested that the prevalence of varicocele is positively associated with age [9], height [3, 8–15], weight [10, 11, 14] and negatively correlated with BMI [3, 7–10, 15–20]. Other studies have suggested that the prevalence of varicocele is negatively associated with age [3] and weight [12, 15] or that the prevalence of varicocele is not associated with age [13, 18, 21], weight [21], height [13, 21, 22], or BMI [11, 13, 21].

There are reports that the severity of varicoceles is inversely correlated with age [20] and BMI [18, 19] or that the severity of varicoceles increases with height [12]. Other studies have reported that the severity of varicoceles is not associated with age [21], weight [12, 21], height [21], and BMI [12, 21]. In addition, Bake et al. [23] reported that patients with grade III varicocele had a lower BMI than those with grade I and II varicocele, but this was not significant.

The objective of this systematic review and meta-analysis was to evaluate the effect of age, height, weight, and BMI on the prevalence and severity of varicocele.

## Materials and methods

### Search strategy

The study was conducted according to the Preferred Reporting Items for Systematic Reviews and Meta-Analyses (PRISMA) guidelines [24]. Databases including EMBASE, MEDLINE, PubMed, Cochrane Library, China National Knowledge Infrastructure (CNKI), Web of Science, and Google Scholar were systematically searched to identify relevant articles published up to March 2020. We searched the literature using the following terms: “varicocele”, “varicoceles”, “varicocelegrade”, “body mass index”, “BMI”, “age”, “height”, “weight”, and “somatometric parameters”. Patient informed consent and ethical approval were not required since this study is a meta-analysis based on published articles.

### Inclusion and exclusion criteria

Observational and experimental studies were included in this systematic review and meta-analysis if they met the following criteria: (1) the topic is varicocele; (2) observational studies published as original studies to assess the effect of age, height, weight, and BMI on the prevalence and/or severity of varicocele; (3) directly measured height and weight; (4) the data for age, height, weight or BMI should be reported as the means with standard deviations (SDs); and (5) sufficient data to calculate the weighted mean differences (WMDs). The exclusion criteria were as follows: (1) abstracts, reviews, letters, and editorials; (2) case-only studies; (3) unpublished or inaccessible full articles; and (4) duplicate publications. Grades of varicocele were determined according to physical examination and sonographic parameters. Varicocele was graded as follows: grade I, palpable only with the Valsalva manoeuvre; grade II, palpable without the Valsalva manoeuvre but not visible; and grade III, visible from a distance without palpation [21].

### Study selection

Two authors (R.L. and J.L.) independently reviewed all articles based on the predetermined inclusion/exclusion criteria, and the results were cross-checked. Relevant articles were initially identified by reviewing the titles and abstracts. When appropriateness could not be determined, the full-text of each remaining article was retrieved and assessed to determine whether the inclusion/exclusion criteria were satisfied. If any disagreements occurred, a third author (Y.L.) reviewed the article and made a final decision after careful discussion.

### Data extraction

Two authors (R.L. and J.L.) independently extracted data from the eligible studies, and the final results were cross-checked. If any disagreements occurred, a third author (Y.L.) reviewed the article and made a final decision after careful discussion. For each eligible study, the following information was collected: author name, year of publication, type of study design, country of origin, ethnicity group, sample size, and age.

### Quality assessment of the included studies

The quality of the included case-control studies was assessed using the Newcastle-Ottawa Scale (NOS) [25]. The quality of the included cross-sectional studies was assessed using the Agency for Healthcare Research and Quality (AHRQ) criteria [26].

### Statistical analysis

Meta-analysis was performed using Stata SE 12.0 software (StataCorp LP, USA). Cochran’s Q statistic and I^2^ statistics were used to assess heterogeneity (*P* < 0.10 and/or *I*^*2*^>50% indicated significant heterogeneity). Random-effects models were used to obtain the pooled WMDs and 95% confidence intervals (CIs). Sensitivity analysis was performed by excluding each study to examine the influence of individual studies on the pooled results. Possible publication bias was assessed using Begg’s funnel plot and Egger’s regression test. *P* < 0.05 was considered statistically significant.

## Results

### Study selection

**Fig. 1:**
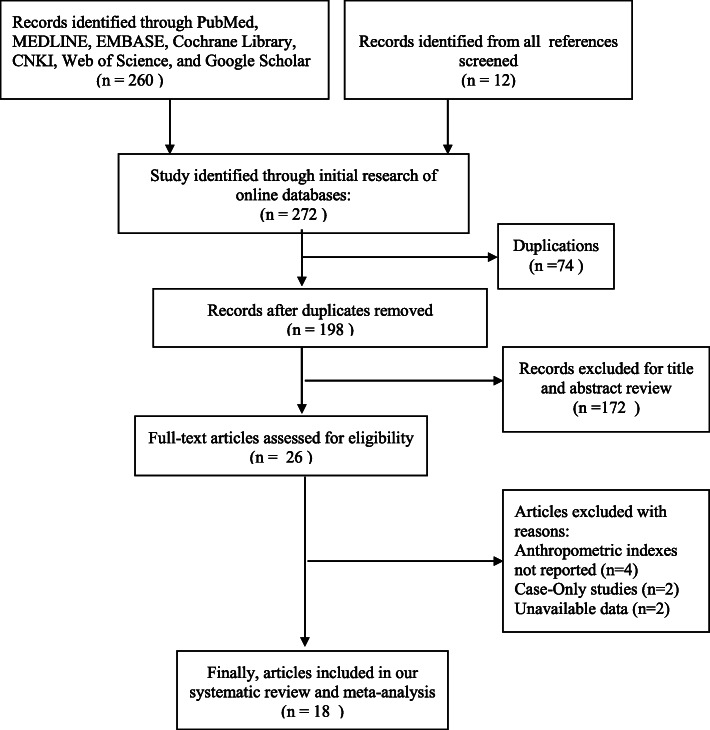
Flow chart of the identified, included, and excluded studies

Details of the search and screening process are graphically described in Fig. 1. Based on our search strategy, 272 studies were identified. After removing 74 duplicate studies, we reviewed the titles and abstracts of 198 studies. After reading the titles and abstracts, 26 studies were included. After reading the full text of the remaining studies, 8 studies were excluded for various reasons. Finally, 18 studies were eligible for the meta-analysis, which involved 1,391,360 subjects (56,390 patients with varicocele and 1,334,970 patients without varicocele).

### Study characteristics and quality

The main characteristics of the included studies are summarized in Table 1. Overall, these included studies were published between 2006 and 2018. Among the 18 studies, 11 were case-control studies, and 7 were cross-sectional studies.

**Table 1:** Characteristics of participants in included studies

Study	Year	Study design	Ethnicity	Country	Sample size		Age (years)	Included varicocele patients	Quality score
					Varicocele	Non-varicocele			
Handel et al. [16]	2006	Case-control	Caucasian	USA	1093	2120	NA	Left. right and bilateral	8
Kilic et al. [13]	2007	Case-control	Caucasian	Turkey	52	100	14–50	Left. right and bilateral	7
Tsao et al. [19]	2009	Cross-sectional	Asian	China	490	560	18–27	Left. right and bilateral	8
Chen et al. [18]	2010	Case-control	Asian	China	102	95	18–50	NA	7
Farhan et al. [27]	2010	Case-control	Asian	Iraq	206	206	NA	Left. right and bilateral	7
Chancwalters et al. [28]	2012	Case-control	Caucasian	USA	330	749	18–40	Left. right and bilateral	7
Soylemez et al. [21]	2012	Cross-sectional	Caucasian	Turkey	498	1563	19–34	Left. right and bilateral	7
Yigitler et al. [29]	2012	Cross-sectional	Caucasian	Turkey	750	11,831	16–23	NA	9
Özçelik et al. [30]	2013	Case-control	Caucasian	Turkey	75	100	20–30	NA	7
Rais et al. [31]	2013	Cross-sectional	Caucasian	Israel	47,398	1,275,663	17–18	NA	9
Gokce et al. [8]	2013	Cross-sectional	Caucasian	Turkey	587	1255	18–50	Right and bilateral	8
Doğantekin et al. [32]	2014	Cross-sectional	Caucasian	T urkey	210	390	21–38	Left. right and bilateral	7
Bae et al. [33]	2014	Case-control	Asian	Korea	211	102	NA	NA	7
Gorur et al. [34]	2015	Case-control	Caucasian	Turkey	138	117	18–45	NA	7
Liu et al. [35]	2015	Case-control	Asian	China	73	104	18–50	NA	7
Shafi et al. [36]	2015	Case-control	Asian	Iran	153	250	18–40	Left. right and bilateral	7
Liu et al. [7]	2017	Cross-sectional	Asian	China	1911	37,648	21–49	Left. right and bilateral	8
Pallotti et al. [3]	2018	Case-control	Caucasian	Italy	2085	2080	NA	Left. right and bilateral	7

### Age of varicocele and nonvaricocele patients

**Fig. 2:**
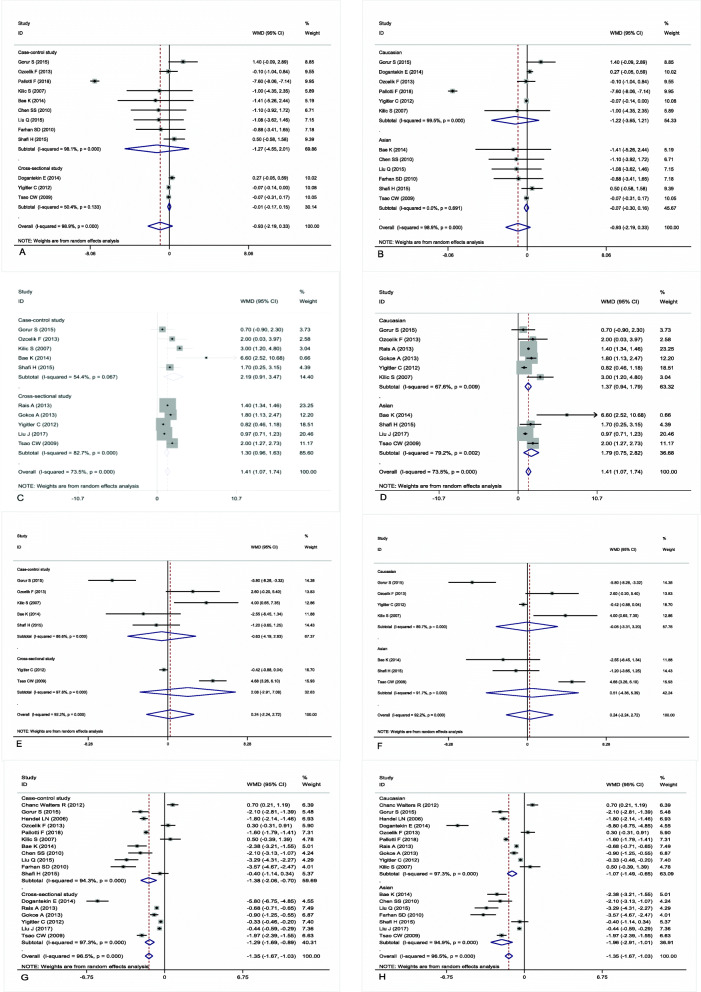
Association between age and varicocele in the random-effects model of observational studies by the type of studydesign (**a**) and ethnicity (**b**); association between height and varicocele in the random-effects model of observational studies by the type of studydesign (**c**) and ethnicity (**d**); association between weight and varicocele in the random-effects model of observational studies by the type of studydesign (**e**) and ethnicity (**f**); association between body mass index (BMI) and varicocele in the random-effects model of observational studies by the type of studydesign (**g**) and ethnicity (**h**)

Twelve studies investigated the relationship between age and the prevalence of varicocele. The overall results showed that there was no association between age and the prevalence of varicocele (WMD = -0.93, 95% CI = -2.19 to 0.33, *P* = 0.149) (Fig. 2a, Fig. 2b, and Table 2).

**Table 2:** Meta-analysis results of age, height, weight, and body mass index (BMI)

Outcomes	*N*	Model used	Heterogeneity		Pooled WMD		Begg’s test *P*
			I^2^ (%)	*P* value	WMD (95 CI)	*P* value	
Age							
Case –control study	9	Random-effects	98.1	0.000	−1.27 (−4.55 to 2.01)	0.448	
Cross-sectional study	3	Random-effects	50.4	0.133	−0.01 (−0.17 to 0.15)	0.902	
Caucasian	6	Random-effects	99.5	0.000	−1.22 (−3.65 to 1.21)	0.325	
Asian	6	Random-effects	0.0	0.691	−0.07 (− 0.30 to 0.16)	0.542	
Overall	12	Fixed-effects	98.9	0.000	−0.93 (−2.19 to 0.33)	0.149	0.446
height							
Case –control study	5	Random-effects	54.4	0.067	2.19 (0.91 to 3.47)	0.001	
Cross-sectional study	5	Random-effects	82.7	0.000	1.30 (0.96 to 1.63)	0.000	
Caucasian	6	Random-effects	67.6	0.009	1.37 (0.94 to 1.79)	0.000	
Asian	4	Random-effects	79.2	0.002	1.79 (0.75 to 2.82)	0.001	
Overall	10	Random-effects	73.5	0.000	1.41 (1.07 to 1.74)	0.000	0.681
weight							
Case –control study	5	Random-effects	86.6	0.000	−0.63 (−4.19 to 2.93)	0.729	
Cross-sectional study	2	Random-effects	97.8	0.000	2.08 (−2.91 to 7.08)	0.414	
Caucasian	4	Random-effects	89.7	0.000	−0.05 (−3.31 to 3.20)	0.975	
Asian	3	Random-effects	91.7	0.000	0.51 (−4.36 to 5.39)	0.837	
Overall	7	Random-effects	92.2	0.000	0.24 (−2.24 to 2.72)	0.850	0.746
BMI							
Case –control study	11	Random-effects	94.3	0.000	−.1.38 (−2.06 to −0.70)	0.000	
Cross-sectional study	6	Random-effects	97.3	0.000	−1.29 (−1.69 to −0.89)	0.000	
Caucasian	10	Random-effects	97.3	0.000	−1.07 (−1.49 to −0.65)	0.000	
Asian	7	Random-effects	94.9	0.000	−1.96 (−2.91 to − 1.01)	0.000	
Overall	17	Random-effects	96.5	0.000	−1.35 (− 1.67 to − 1.03)	0.000	0.097

### Height of varicocele and nonvaricocele patients

Ten studies investigated the relationship between height and the prevalence of varicocele. The overall results showed that patients with varicocele were significantly taller than patients without varicocele (WMD = 1.41, 95% CI = 1.07 to 1.74, *P* < 0.001) (Fig. 2c, Fig. 2d, and Table 2). However, there was between-study heterogeneity that could not be ignored (*I*^*2*^ = 73.5%, *P* < 0.001). Therefore, stratified analyses by study design and ethnicity were performed to explore the origin of significant heterogeneity. In the subgroup analysis of study design, patients with varicocele were significantly taller than patients without varicocele in the case-control studies (WMD = 2.19, 95% CI = 0.91 to 3.47, *P* = 0.001) and cross-sectional studies (WMD = 1.30, 95% CI = 0.96 to 1.63, *P*<0.001) (Fig. 2c and Table 2). Similarly, the subgroup analysis by ethnicity indicated that patients with varicocele were significantly taller than patients without varicocele in the Asian population (WMD = 1.79, 95% CI = 0.75 to 2.82, *P* = 0.001) and Caucasian population (WMD = 1.37, 95% CI = 0.94 to 1.79, *P* < 0.001) (Fig. 2d and Table 2).

### Weight of varicocele and nonvaricocele patients

Seven studies investigated the relationship between weight and the prevalence of varicocele. The overall results showed that there was no association between weight and the prevalence of varicocele (WMD = 0.24, 95% CI = -2.24 to 2.72, *P* = 0.850) (Fig. 2e, Fig. 2f, and Table 2).

### BMI of varicocele and nonvaricocele patients

Seventeen studies investigated the relationship between BMI and the prevalence of varicocele. The overall results showed that patients with varicocele had a significantly lower BMI than patients without varicocele (WMD = -1.35, 95% CI: − 1.67 to − 1.03, *P* < 0.001) (Fig. 2g, Fig. 2h, and Table 2). However, there was between-study heterogeneity that could not be ignored (*I*^*2*^ = 96.5%, *P* < 0.001). Therefore, stratified analyses by study design and ethnicity were performed to explore the origin of the significant heterogeneity. In the subgroup analysis of study design, patients with varicocele had a lower BMI than patients without varicocele in the case-control studies (WMD = -1.38, 95% CI = -2.06 to − 0.70, *P*<0.001) and cross-sectional studies (WMD = -1.29, 95% CI = -1.69 to − 0.89, *P*<0.001) (Fig. 2g and Table 2). Similarly, the subgroup analysis by ethnicity indicated that patients with varicocele had a lower BMI than patients without varicocele in the Asian population (WMD = -1.96, 95% CI = -2.91 to − 1.01, *P* < 0.001) and Caucasian population (WMD = -1.07, 95% CI = -1.49 to − 0.65, *P* < 0.001) (Fig. 2h and Table 2).

### BMI of patients with different grades of varicocele

**Fig. 3:**
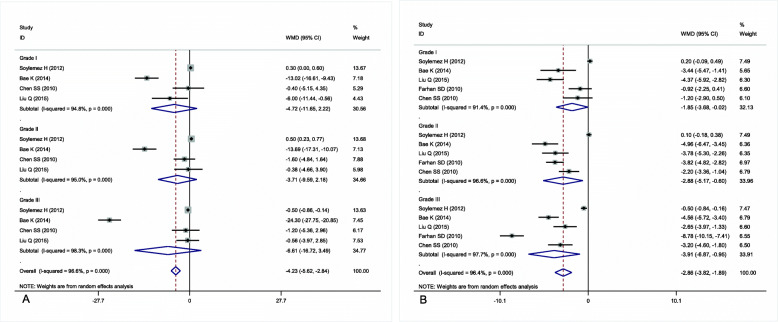
**a** Forest plot of the association between age and severity of varicocele; **b** forest plot of the association between body mass index (BMI) and severity of varicocele

**Fig. 4:**
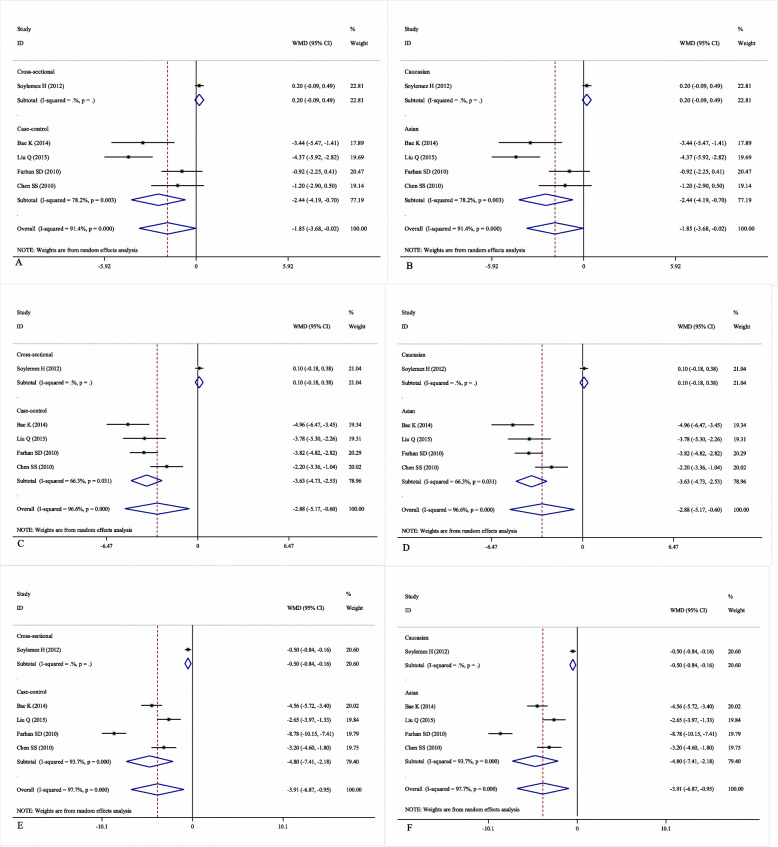
Association between body mass index (BMI) and grade I varicocele in the random-effects model of observational studies by the type of study design (**a**) and ethnicity (**b**); association between body mass index (BMI) and grade II varicocele in the random-effects model of observational studies by the type of study design (**c**) and ethnicity (**d**); association between body mass index (BMI) and grade III varicocele in the random-effects model of observational studies by the type of study design (**e**) and ethnicity (**f**)

We performed a subgroup analysis to investigate the effect of BMI on the severity of varicocele***.*** Patients with grades I, II, and III varicocele had a lower BMI than patients without varicocele, with WMDs of − 1.85 (95% CI: − 3.68 to − 0.02), − 2.88 (95% CI: − 5.17 to − 0.60), and − 3.91 (95% CI: − 6.87 to − 0.95), respectively (Fig. 3b). The grade of varicocele was inversely correlated with increased BMI. However, there was between-study heterogeneity that could not be ignored (*I*^*2*^ = 96.4%, *P*<0.001). Therefore, stratified analyses by study design and ethnicity were performed to explore the origin of the significant heterogeneity. In the subgroup analysis of study design, patients with grade I varicocele had a lower BMI than patients without varicocele in the case-control studies (WMD = -2.44, 95% CI = -4.19 to − 0.70, *P* = 0.003) (Fig. 4a). Similarly, the subgroup analysis by ethnicity indicated that patients with grade I varicocele had a lower BMI than patients without varicocele in the Asian population (WMD = -2.44, 95% CI = -4.19 to − 0.70, *P* = 0.003) (Fig. 4b). In the subgroup analysis of study design, patients with grade II varicocele had a lower BMI than patients without varicocele in the case-control studies (WMD = -3.63, 95% CI = -4.73 to − 2.53, *P* = 0.031) (Fig. 4c). Similarly, the subgroup analysis by ethnicity indicated that patients with grade II varicocele had a lower BMI than patients without varicocele in the Asian population (WMD = -3.63, 95% CI = -4.73 to − 2.53, *P* = 0.031) (Fig. 4d). In the subgroup analysis of study design, patients with grade III varicocele had a lower BMI than patients without varicocele in the case-control studies (WMD = -4.80, 95% CI = -7.41 to − 2.18, *P*<0.001) (Fig. 4e). Similarly, the subgroup analysis by ethnicity indicated that patients with grade I varicocele had a lower BMI than patients without varicocele in the Asian population (WMD = -4.80, 95% CI = -7.41 to − 2.18, *P*<0.001) (Fig. 4f).

### Sensitivity analyses

**Fig. 5:**
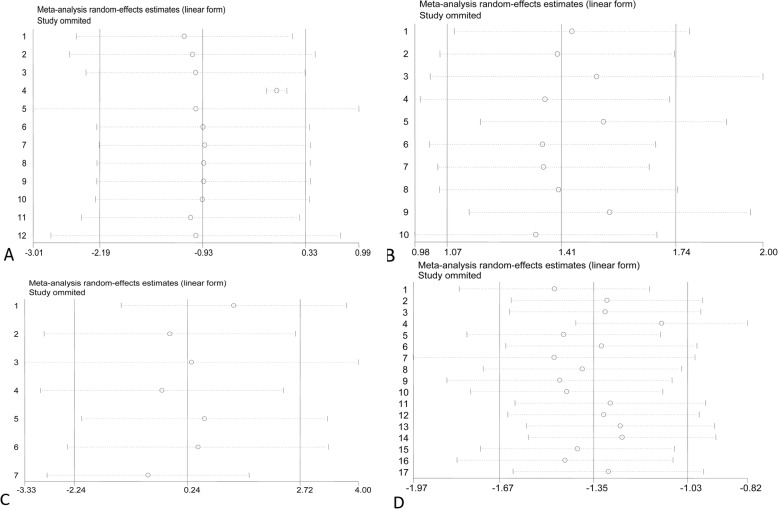
**a** Sensitivity analysis for the association between age and varicocele by random-effects analysis; **b** sensitivity analysis for the association between height and varicocele by random-effects analysis; **c** sensitivity analysis for the association between weight and varicocele by random-effects analysis; **d** sensitivity analysis for the association between body mass index (BMI) and varicocele by random-effects analysis

Sensitivity analysis was conducted by excluding each study to evaluate possible biases. Our results showed that the overall effects did not significantly change after excluding any one study, indicating that the meat-analysis results were stable and reliable (Fig. 5).

### Publication bias

**Fig. 6:**
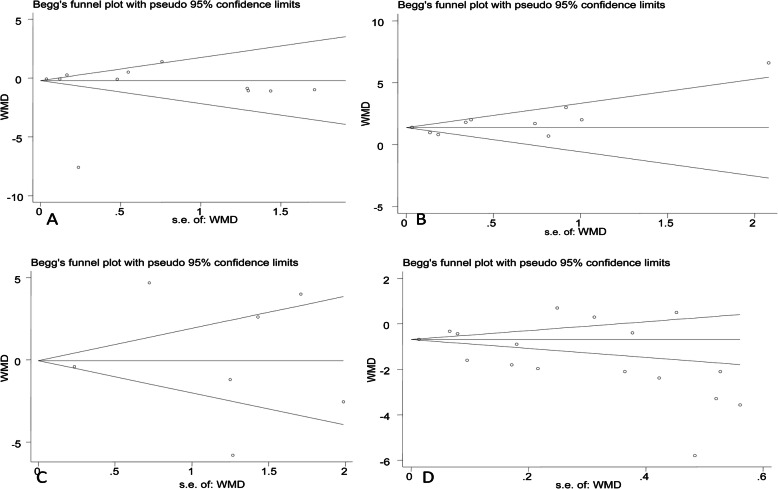
**a** Begg’s funnel plot and pseudo 95% confidence intervals (CIs) of 12 studies included in the meta-analysis of studies regarding publication bias for age; **b** Begg’s funnel plot and pseudo 95% confidence intervals (CIs) of 10 studies included in the meta-analysis of studies regarding publication bias for height; **c** Begg’s funnel plot and pseudo 95% confidence intervals (CIs) of 7 studies included in the meta-analysis of studies regarding publication bias for weight; **d** Begg’s funnel plot and pseudo 95% confidence intervals (CIs) of 17 studies included in the meta-analysis of studies regarding publication bias for body mass index (BMI)

Begg’s funnel plot and pseudo 95% CIs are presented in Fig. 6. Egger’s test did not show any publication bias for age (*P* = 0.446), height (*P* = 0.681), weight (*P* = 0.746), or BMI (*P* = 0.097).

## Discussion

Varicocele, the most common cause of male infertility, can impair spermatogenesis. The exact mechanism of varicocele development has not been fully clarified, although the “nutcracker phenomenon” theory has been widely accepted. The “nutcracker phenomenon” refers to the compression of the left renal vein between the superior mesenteric artery and the abdominal aorta [37].

Some studies have reported that there are no significant differences in age between patients with and without varicocele [13, 18, 19, 21]. Prabakaran et al. [9] and Pallotti et al. [3] reported that the incidence of varicocele was positively correlated with age. Al-Ai et al. [20] reported that age was inversely associated with varicocele grade. In this meta-analysis, we found that age was not associated with the prevalence or severity of varicocele.

Some studies have reported that there are no significant differences in height between patients with and without varicocele [13, 21, 22], whereas other studies have reported that patients with varicocele are significantly taller than patients without varicocele [3, 8–11, 14, 19]. The pooled results demonstrated that patients with varicoceles were taller than patients without varicoceles. That is to say, shorter height protects against varicocele and is associated with a decreased incidence of varicocele. Tsao et al. [19] speculated that taller height may be related to increased hydrostatic pressure in the spermatic vein, which in turn overwhelms the valve mechanisms in the veins, resulting in the formation of varicocele. Stratified analyses were performed to explore the influence of study design and ethnicity. We found a taller height in patients with varicocele in both Asian and Caucasian populations. We also found a taller height in patients with varicocele in both case-control study and cross-sectional study.

Some studies have reported that the weight of patients with varicocele is significantly heavier than that of those without varicocele [10, 11, 13, 14]. Kumanov et al. [15] and Tsao et al. [19] reported that the weight of patients with varicocele was significantly lighter than that of those without varicocele. Soylemez et al. [21] reported that there was no significant difference in weight between patients with and without varicocele. In this meta-analysis, we found that weight was not associated with the incidence of varicocele.

Controversial findings on the association between BMI and varicocele have been reported in the literature. Some studies have reported that there is no significant differences in BMI between patients with and without varicocele [11, 13, 21], whereas other studies have reported that the prevalence of varicocele is inversely correlated with BMI [3, 8–10, 12, 15–20]. In this study, the pooled results demonstrated that patients with varicocele had lower BMI than patients without varicocele. Thus, higher BMI protects against varicocele and is associated with a decreased incidence of varicocele. Our results support the “nutcracker phenomenon” theory. Subgroup analyses were performed to explore the influence of study design and ethnicity. We found a lower BMI in patients with varicocele in both Asian and Caucasian populations. We also found a lower BMI in patients with varicocele in both case-control and cross-sectional studies. The association between BMI and the incidence of varicocele is due to a reduced nutcracker phenomenon in overweight and obese men.

Some studies [12, 21, 33, 35] have reported that BMI does not affect the severity of varicocele. Chen et al. [18] reported that patients with grade III varicocele had a lower BMI than patients with grade I and II varicocele, but the difference was not significant. Farhan et al. [27] reported that varicocele grade significantly decreased with increasing BMI. In this meta-analysis, we found that the grade of varicocele was inversely correlated with increased BMI.

There were three particular strengths of this systematic review and meta-analysis. First, 56,325 patients with varicocele and 1,334,694 patients without varicocele were included in the meta-analysis. Therefore, the sample size was large enough to draw a reliable conclusion. Second, we performed stratified analyses by study design and ethnicity. Third, we investigated the effect of age and BMI on the severity of varicocele.

There were two limitations to this meta-analysis. First, heterogeneity among studies still existed although we applied strict inclusion and exclusion criteria. Second, the number of included studies was small for some subgroups.

We found that the prevalence of varicocele was significantly associated with height and inversely correlated with BMI. The severity of varicocele was inversely correlated with increased BMI. Our results remind us of the necessity of early screening and treatment for varicocele in taller men and underweight men.

## Data Availability

The datasets used during the current study are available from the corresponding author on reasonable request.

## Abbreviations

BMI: Body mass index
CNKI: China National Knowledge Infrasturcture
WMDs: Weighted mean differences
CIs: Confidence intervals
SDs: Standard deviations
